# 

**Published:** 1850-12

**Authors:** 


### NOTICE TO CORRESPONDENTS.

Communications and Books for notice should be addressed to the Editor, care of Messrs. Lindsay & Blakiston.

Letters, &c., connected with the *business affairs* of the Journal should be addressed to the Publishers.

Papers for publication must be received *before* the 20th of the month, or they cannot appear in the forthcoming number.

The following Journals have been received in exchange:

American Journal of Medical Sciences. Oct.
Western Journal.
New Orleans Journal.
Charleston Medical Journal and Review.
North Western Medical and Surgical Journal.
New York Journal of Medicine and Collateral Sciences.
Nordamerikanischer monatsbericht für Natur-und Heilkunde.
New York Medical Gazette. Weekly.
The Boston Medical and Surgical Journal. (Weekly, Boston.)
Buffalo Journal.
Medical News.
American Journal of Insanity.
Southern Medical and Surgical Journal. Oct.
The London Lancet. (Weekly, London.)
The Medical Times. Weekly, London.
Dublin Medical Press.
British and Foreign Medico-Chirurgical Review. Oct.
Provincial Medical and Surgical Journal.
London Medical Gazette. Sept. and Oct.
London Medical Examiner. Sept., Oct. and Nov.
Pharmaceutical Journal. (London.)
Edinburgh Medical and Surgical Journal. Oct.
Journal of Psychological Medicine. Oct.
Monthly Journal of Medical Science. (Edinburgh.) Oct. and Nov.

—

The following works have also been received for notice:

Howard on the Eye.
Spectacles, their Uses and Abuses. By Sichel.
An Essay on Zoo-Adynamia. By J. G. Ziegler, M. D., Philadelphia.
Woman and her Diseases, by C. D. Meigs, M. D. 2d edition. From Lea & Blanchard.
Diseases and Surgical Operations of the Mouth, by Jourdain. From Lindsay & Blakiston.
Diseases of the Kidneys, by F. A. Ramsay, M. D.
Chart of Auscultation and Percussion, by C. Claiborne Gooch, M. D., Richmond, Va.

—

Communications have been received from:—

W. J. Reese, M. D., Alabama.
William Waters, M. D., Fredericktown, Md.
G. H. H. Koontz, Woodstock, Va.
E. C. Banks, Lawrenceville, Ill.
J. Travis, M. D., Tennessee.

—

The foreign correspondents of the Examiner will please direct their Exchanges and other communications to the care of Mr. Charles J. Skeet, 27 King William St., Charing Cross, London, or Mr. H. Bossange, 21 Bis, Quai Voltaire, Paris

